# Dynamic Post-Transcriptional Regulation of HIV-1 Gene Expression

**DOI:** 10.3390/biology1020116

**Published:** 2012-07-03

**Authors:** Anna Kula, Alessandro Marcello

**Affiliations:** Laboratory of Molecular Virology, International Centre for Genetic Engineering and Biotechnology (ICGEB), Padriciano, Trieste 99 34012, Italy; Email: kula@icgeb.org

**Keywords:** HIV-1, RNA, transcription, splicing, Rev

## Abstract

Gene expression of the human immunodeficiency virus type 1 (HIV-1) is a highly regulated process. Basal transcription of the integrated provirus generates early transcripts that encode for the viral products Tat and Rev. Tat promotes the elongation of RNA polymerase while Rev mediates the nuclear export of viral RNAs that contain the Rev-responsive RNA element (RRE). These RNAs are exported from the nucleus to allow expression of Gag-Pol and Env proteins and for the production of full-length genomic RNAs. A balance exists between completely processed mRNAs and RRE-containing RNAs. Rev functions as an adaptor that recruits cellular factors to re-direct singly spliced and unspliced viral RNAs to nuclear export. The aim of this review is to address the dynamic regulation of this post-transcriptional pathway in light of recent findings that implicate several novel cellular cofactors of Rev function.

## 1. Introduction

Replication of retroviruses including the Human immunodeficiency virus type 1 (HIV-1) occurs in the nucleus of infected cells. The incoming viral RNA genomes are reverse transcribed in the cytoplasm and the resulting DNA transported to the nucleus where it is integrated into chromatin [1,2]. The chromatinized provirus becomes part of the host cell’s genome and is subjected to the complex network of regulatory pathways that control cellular gene expression [3,4]. The understanding of HIV-1 gene expression has profound pathological implications. Persistence of HIV-1 infection in patients undergoing anti-retroviral therapy is the major barrier to viral eradication [5,6,7]. Treatment intensification strategies have shown that viremia could originate both from virus replicating in sanctuaries, where drug levels are low, and from latently infected cells [8,9,10,11]. These long-lived cells harbor a provirus unable to express its genes and, as such, invisible to the immune system and to antiviral drugs. Therefore a better understanding of the specific features of HIV-1 gene expression will help in designing alternative therapies aimed at the eradication of these persistent viral reservoirs [12].

Three features distinguish the HIV-1 provirus from the host genes: (i) cis-acting DNA/RNA elements present in the provirus/viral RNA; (ii) the activity of the viral trans-activator of transcription Tat and (iii) the activity of the viral regulatory protein Rev. All other factors required for efficient viral transcription and RNA processing are of cellular origin, including the RNA polymerase (RNAPII). The generation of infectious retroviral progeny requires the synthesis and export to the cytoplasm of spliced subgenomic mRNAs for protein translation, of partially spliced RNAs that function as the mRNA for the viral proteins Gag-Pol and Env and of the viral genomic RNA [13,14]. Hence, coordinated expression of these three classes of RNAs is a hallmark of HIV-1 efficient replication. Our current understanding is that cellular transcription factors control basal transcription from the viral long terminal repeat (LTR) promoter while the viral Tat protein releases RNAPII stalled after the trans-activator response (TAR) RNA element, present at the 5’end of all transcripts and promotes RNAPII elongation [15,16]. Tat binds TAR and functions as an adaptor of the Cyclin T1/CDK9 kinase complex and for a number of cellular factors involved in the fine-tuning of the process [17,18]. Rev promotes the export of RNAs from the nucleus through the association to an RNA element called the Rev response element (RRE) that is present in the env gene [19,20,21]. Nuclear export occurs upon association of the Rev with the nuclear export factor Exportin 1 (Crm-1) and translocation of the Rev/RNA complex to the cytoplasm where it is either translated or packaged into virions [22,23,24,25]. The focus of this review will be on the journey of viral RNA from the site of viral transcription to the nuclear pore and on the role of Rev and cellular co-factors in determining the fate of HIV-1 RNA. 

## 2. Spatial and Temporal Definition of HIV-1 Transcription

**Figure 1:**
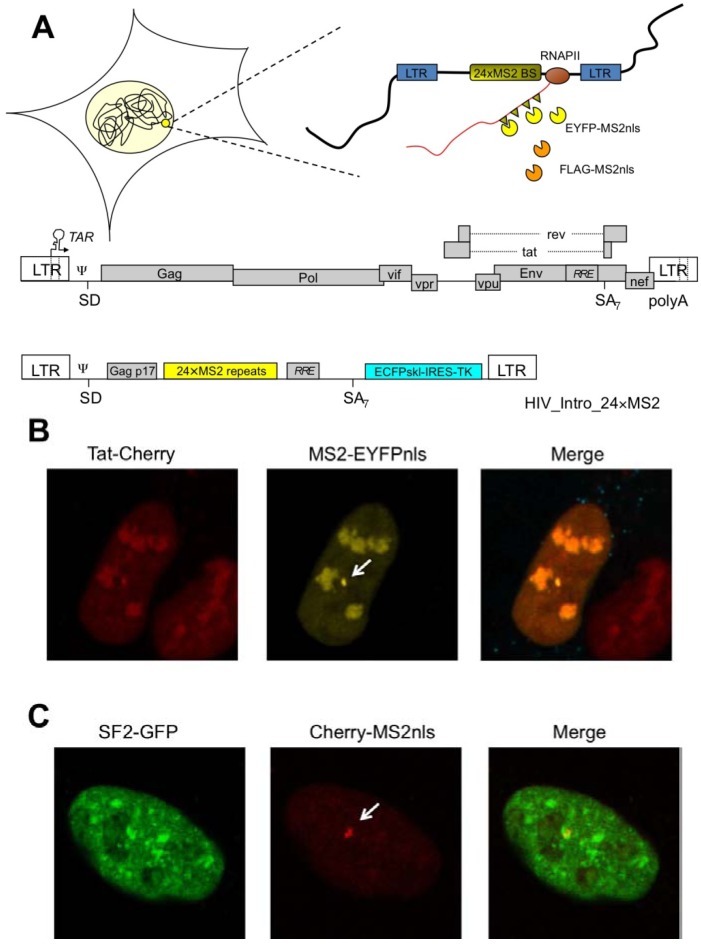
(**a**) Schematic description of the HIV-1 MS2-tagging method. Top, the viral cassette carrying the MS2 repeats is integrated in the cell’s chromatin. RNAPII transcribes along the provirus and produces tagged RNAs that are bound by EYFP-MS2nls for visualization of the nascent RNA. Alternatively, FLAG-MS2nls can be used to pull-down the viral RNA for affinity purification of the associated proteome. An outline of the full-lengthviral genome is also shown below with the construct HIV_intro_24xMS2 (not drawn to scale). These constructs are described in great detail in a series of papers [30,32,35]; (**b**) Localization of Tat on HIV-1 RNA at the transcription site. Tat-Cherry was found associated with the transcription site marked by the accumulation of MS2 (white arrow) in U2OS cell clones expressing EYFP-MS2nls; (**c**) Localization of SF2 on HIV-1 RNA at the transcription site. SF2-GFP was found associated with the transcription site marked by the accumulation of MS2 (white arrow) in U2OS cell clones expressing Tat and EYFP-MS2nls.

HIV-1 RNA starts its journey at the provirus integration site, where RNAPII transcribes the viral genome, and reaches the nuclear pore to exit the nucleus. HIV-1 integrates with high frequency within active genes, possibly because of a better accessibility of open chromatin [26]. Investigation of the spatial positioning of the provirus in latently infected cells demonstrated a preference towards the heterochromatic periphery of the nucleus [27]. Upon reactivation, the transcribing provirus maintains its position in the proximity of the nuclear membrane. Therefore, the pre-integration complex targets a subset of active genes at the nuclear periphery that are outside the heterochromatic regions, tightly associated to the nuclear lamina and are possibly associated to the nuclear pore [28]. These studies on the topology of HIV-1 transcription were conducted by three-dimensional in situ hybridization and by a novel technique that permits visualization of viral RNA in living cells [29]. Taking advantage of the MS2 phage core protein and its high-affinity RNA binding site it has been possible to engineer cell lines carrying an integrated provirus where arrays of MS2 binding sites were cloned in the viral genome [30,31,32]. As depicted in Figure 1A, MS2 fused to an autofluorescent protein like EYFP (enhanced yellow fluorescent protein) and to a nuclear localization signal (nls) would recognize nascent HIV-1 RNA. At steady state in a live cell the nucleus would appear yellow with a spot corresponding to the HIV-1 transcription site. This is clearly visible in Figure 1B, where HIV-1 transcription is induced by a hybrid Tat fused to the Cherry autofluorescent protein. As expected, Tat-Cherry is also present at the transcription site on nascent RNA. With this tool it has been possible to study not only the sub-nuclear localization of the transcribing provirus, but also the dynamic recruitment of factors like Tat, CDK9 or RNAPII as well as the kinetics of RNAPII transcription [30,31,33]. Recently, the generation of cell lines transduced by a HIV vector carrying the MS2 repeats allowed for the first time the analysis of RNAPII transcription rates on a single Tat-induced transcribing provirus localized at the nuclear periphery [33]. By photobleaching techniques it was shown that RNAPII could elongate at least an order-of-magnitude faster than previously thought. These data were confirmed by the quantification of single (spliced) RNAs per cell at steady state reaching the conclusion that the output rate of HIV-1 transcription was of one new RNA every 1.2–2.4 seconds. These studies demonstrate that Tat-mediated HIV-1 transcription is highly efficient and able to produce a large amount of pre-mRNA in a short time [34]. But how is the downstream processing controlled in order to produce the large variety of RNA species characteristic of HIV-1?

## 3. A Portfolio of HIV-1 RNA Species

After RNA synthesis, the HIV-1 transcript is processed through alternative splicing. The transcripts are grouped into three size classes: the unspliced, 9 kb RNA that encodes Gag and Gag/Pol but also forms the genomic RNA, the 4 kb, singly spliced RNAs that encode Vif, Vpr, Vpu and Env, and the 2 kb, fully spliced RNAs that express Tat, Rev and Nef. Generation of the various viral RNA species is achieved through the use of five 5’ splice donors (SD_1–5_) and nine 3’ splice acceptors (SA_1–9_) [14,36,37]. HIV exploits a variety of mechanisms to make sure that all required RNAs are balanced for an efficient life cycle. Sub-optimal splice acceptor sequences and exon regulatory sequences like exon splicing enhancers (ESE) and exonic/intronic splicing silencers (ESS/ISS) and their associated factors are the players of alternative splicing control [38,39,40,41,42,43,44,45,46]. Factors that promote splicing are, for example, SF2/ASF that binds to ESEs and SC35 while association of hnRNP A1 to ESE/ISS inhibits splicing. This mechanism allows the production of both fully spliced mRNAs as well as unspliced and partially spliced RRE-containing RNAs. The latter are retained in the nucleus and require Rev action on RRE to be exported. HIV-1 also encodes for regulatory sequences called the instability (INS) or *cis*-acting repressor (CRS) sequences [47,48,49,50,51,52]. INS elements impair mRNA stability, nucleo-cytoplasmic transport and translation and are counteracted by the action of Rev. However Rev is unable to export an RRE-containing RNA that does not contain also a functional INS, implying their involvement in a pathway of nuclear RNA processing that is exploited by Rev [51,53]. Several cellular factors like the poly(A) binding protein 1 (PABP1), the heterogenous ribonuclear protein A1 (hnRNP), and the polypyrimidine tract-binding protein associated binding factor PSF and associated factor p54^nrb^ complex, were shown to bind specifically to INS elements [53,54,55,56]. One possible mechanism is that these INS-binding factors divert INS/RRE containing HIV-1 RNAs from the splicing machinery and promote nuclear retention until Rev takes care of their export from the nucleus [56]. Although this hypothesis has been around since the discovery of INS sequences, there is still not a definitive answer on the intranuclear fate of unspliced HIV-1 RNAs and on the cellular factors that are involved in this pathway.

## 4. HIV-1 Rev & Friends

Rev is an essential HIV-1 protein encoded from two exons that are joined by splicing to produce a monocistronic transcript, early in the viral replication cycle [57,58]. In this chapter the focus will be on Rev-host factors interactions (Table 1), other aspects of Rev have been described in excellent reviews and will not be discussed in detail here [22,23,24,25]. Briefly, Rev is a small (19 kD), protein of approximately 116 amino acids in size that contains a basic arginine-rich RNA binding domain functioning also as a nuclear localization sequence (NLS), a leucine-rich nuclear export signal (NES) that binds Crm-1 (see below) and a protein self-multimerization domain. Rev is post-translationally modified by phosphorylation and possibly methylation [59,60]. Rev is a nuclear shuttling protein that specifically recognizes an RNA element located within the coding sequence of Env. This sequence, called the RRE (Rev-responsive element), forms a highly structured secondary RNA structure. Rev binds to this region through the basic arginine-rich domain. Binding of Rev to RRE is initiated first by low-affinity interaction of a single Rev monomer, followed by the cooperative binding of several Rev molecules to the RRE. Although Rev is best known to stimulate nucleocytoplasmic transport of incompletely spliced viral RNAs, other activities in translation and encapsidation, as well as integration, have been described [61,62,63].

**Table 1:** Cellular cofactors that modulate Rev function.

Cellular Protein	Proposed function in Rev Activity	References
*Following the Order of Their Mention in the Text.*		
CK2	Interacts with and phosphorylates Rev	[59]
PRMT6	Methylates Rev	[60]
Crm-1	Nuclear export or Rev-bound RRE containing RNAs	[64]
Importin-β	Import of Rev into the nucleus	[65,66]
DDX-3	Helicase that promotes nuclear export of Rev-containing RNAs	[67]
DDX-1	Helicase similar to DDX-3 but restricted to astrocytes	[68]
DDX-9 (RHA)	Helicase involved in remodeling RNA upstream of Rev	[69]
DDX-24	Helicase that binds Rev and is involved in viral RNA packaging	[70]
B23	Binds Rev and colocalizes to nulceoli	[71]
p32	Human homologue of yeast YL2	[72]
NAP-1	Interacts with Rev nls	[73]
HIC	Binds Rev in the cytoplasm and modulates nuclear import through nls binding	[74]
eIF-5A	Binds Rev and is involved in nuclear export of viral RNA	[75,76]
PABP1	Associates with HIV-1 RNAs in a Rev-dependent manner	[77]
hRIP	Binds Rev and promotes release of RNA from the nucleus	[78,79]
Sam68	Complements Rev activity	[80,81]
Hax-1	Inhibits Rev function in the cytoplasm	[82]
*Following the Order of Their Mention in the Text.*		
PIMT	5’-CAP modification of Rev-dependent transcripts	[62]
MATR3	Promotes the nuclear export of Rev-dependent RNAs	[35,83]
SF2/ASF	Binds RRE in a Rev-dependent manner	[84]
*Not Described in the Text.*		
RREBP49	hnRNP F homologue that binds RRE	[85]
Pur α	Interacts with Rev and RRE	[86]
Prothymosin α	Interacts with Rev	[87]
NF90	Inhibits Rev function	[88]
IkB	Negatively regulates Rev function	[89]
hnRNPA1	Binds to repressor sequences in gag and stimulates Rev function	[53]
16.4.1	Interacts with Rev and Crm-1	[90]
ATM	Enhances Rev function and viral replication	[91]
β-actin	Involved in Rev activity on nuclear export, together with eIF-5A, or translation	[92,93]

Rev exports the RRE-containing HIV-1 mRNAs from the nucleus through interaction with Crm-1 (chromosome maintenance region 1) [94]. Crm-1 is a member of the karyopherin family of nucleocytoplasmic-transport factors. Crm-1, like other karyopherins involved in nuclear export, binds its cargo in the nucleus in the presence of the GTP-bound form of the Ran GTPase. After nuclear export, hydrolysis of the bound GTP to GDP causes a conformational shift that induces cargo release in the cytoplasm, thus providing the directionality of this export pathway. Crm-1 also interacts with components of the nuclear pore complex (NPC) and this interaction is essential for nuclear RNA export. It is known that Crm-1 is the crucial nuclear export factor for U snRNAs, rRNAs and tRNAs [64]. Their nuclear export is mediated by adaptor proteins bearing leucine-rich NESs similar to the prototypic NES first defined in Rev. A few human mRNAs might also be targeted for export through Crm-1 [95]. In the nucleus, Ran-GTP bound Crm-1 binds the NES domain of Rev, which in turn is bound to RRE-containing HIV-1 transcripts. This interaction enables Crm-1 to export the resulting RNA/protein complex into the cytoplasm, presumably through the Crm-1 connection with Nup214 and Nup98 nucleoporins. In the cytoplasm, conversion from Ran-GTP to Ran-GDP releases the Rev/RNA cargo. Rev then returns to the nucleus by binding to importin-β and Ran-GDP for subsequent rounds of export [65,66].

The Rev-RRE-Crm-1 complex also engages the activity of the cellular RNA helicase DDX3 that functions to enhance the Rev-dependent pathway and it is believed to facilitate the passage of large unspliced HIV-1 RNAs through the nuclear pore [67]. RNA helicases are a large family of cellular proteins involved at various steps of the HIV-1 life cycle [96]. Among them, DDX1 was shown to have an activity similar to DDX3 but limited to assisting Rev activity in human astrocytes [68,97]. Another helicase, RHA (DDX9), has been previously proposed to act upstream of Rev by remodeling the RRE-containing viral RNAs before completion of splicing, thus freeing them for Rev-mediated nuclear export [69]. Finally, DDX24 was shown to bind Rev and to be involved in viral RNA packaging [70].

The nucleolar protein B23 associates tightly with Rev through the basic domain [71]. Rev and B23 colocalize in the nucleoli and the permanence of Rev at that location depends on continuous preribosomal RNA transcription [98]. There have been several reports of cellular interactors of the Rev basic domain. For example, p32, a splicing factor that co-purifies with SF2/ASF, was shown to bind Rev [72] and to overcome an important post-transcriptional block to HIV replication in murine cells [99]. The nucleosome assembly protein 1 (NAP1) was co-purified with Rev and increased its nuclear import [73]. Finally, the Human I-mfa domain-Containing protein (HIC) was reported to regulate Rev nuclear import. HIC selectively blocked importin-β but not transportin-mediated Rev nuclear import via the intermolecular masking of the Rev NLS [74]. The identification of cellular factors that interact with the basic domain of Rev should be handled with great care, particularly concerning specificity. In fact, all these interactors: B23, p32, NAP1, HIC were also shown to interact with the basic domain of Tat (unpublished observations) [100,101,102,103,104]

A number of other interactions have been investigated (Table 1). Some of them were reported only once, others investigated in more detail. The Eukaryotic initiation factor-5A (eIF-5A) was shown to be a cofactor involved in Rev-mediated nuclear export [75,76]. The eIF-5A interacts with specific nucleoporins and is required for efficient interaction of Rev with Crm-1 [93]. The poly(A)-binding protein 1 (PABP1) was shown to associate with cytoplasmic HIV-1 RNAs in a Rev-dependent manner implicating Rev in translation [77]. The human Rev interacting protein (hRIP) was identified by yeast two-hybrid screen as a Rev associated factor [78,79]. The hRIP is an essential HIV cofactor required for HIV replication that promotes the release of incompletely spliced HIV-1 RNAs from the perinuclear region [105,106]. Sam68 (Src-associated protein in mitosis) was shown to be a functional homologue of Rev, able to complement its function in RRE-mediated export of RNA [80]. Sam68 synergizes with Rev in RRE-mediated gene expression and viral production [81]. It is not clear how Sam68 functions and why HIV-1 needs Rev when Sam68 alone can export RRE-containing RNAs. A partial answer has come from the observation that a partner of Sam68, the HS1-associated protein X-1 (Hax-1), interacted with Rev in the cytoplasm inhibiting its activity while Sam68 counteracted this effect [82].

HIV RRE-dependent RNAs are modified by the peroxisome proliferator-activated receptor-interacting protein with methyltransferase domain (PIMT) that induces a modification of the 7-methylguanosine (m7G) cap to create a trimethylguanosine (TMG)-capped RNA [62]. Like cellular small nuclear/nucleolar RNA (snRNA/snoRNA) and telomerase RNA, TMG-capped RNAs are exported from the nucleus through the Crm-1 pathway. Hence, TMG capping may represent another regulatory mechanism for selective expression of HIV-1 genes. 

A recent novel approach to identify HIV-1 RNA binding factors exploited the MS2-tagging of RNA method to affinity purify the ribonucleprotein complex in the cell nucleus [35]. Proteins identified by mass spectrometry included the matrix-associated RNA binding protein Matrin 3 (MATR3) that was shown to interact with Rev through RRE and to be required for Rev-mediated export of RRE-containing HIV-1 RNAs. These observations were also independently confirmed by the group of Jeang [83]. Intriguingly, MATR3 was shown to form a complex with another protein identified in the screen: the polypyrimidine tract-binding protein associated binding factor PSF. MATR3 and PSF have been implicated in the nuclear retention of certain hyperedited RNAs [107]. However, PSF was already implicated in Rev-mediated export of HIV-1 RNAs by its association with the INS RNA elements that control stability of the viral RNA [56]. In addition, MATR3 has been also identified as a constituent of the nuclear pore proteome [108]. Hence, it is tempting to speculate that Rev works with MATR3 to free mRNA from INS-mediated retention through PSF, allowing export through the nuclear pore. 

Rev appears to be a versatile adaptor of cellular factors on RRE-containing HIV-1 RNAs and recent literature has provided attempts to identify the interactome of Rev. Of note the work of Gerace and collaborators who carefully designed a purification scheme to dissect Rev complexes assembled *in vitro* with nuclear or cytosolic extracts in conditions emulating intracellular environments of Rev, where the association of functionally relevant binding factors could be predicted to be different [109]. Reassuringly, several of the already described partners of Rev were found in this analysis including Crm-1, MATR3, NAP-1, DDX24 and DDX1. On the contrary, the monumental work by Nevan Krogan and collaborators who attempted to map the global interactome of HIV-1 proteins in infected cells provided only a handful of validated hits for Rev [110]. Of note is the stringent criterium for validation that included parallel pulldowns of hits with Rev and Tat as control of specificity. None of these hits were previously identified as Rev interactors. Reasons for this low efficiency may rely on the timing of cell harvest, since Rev is an early viral protein, or on the relative abundance of Rev in the infected cell.

## 5. From Transcription to Nuclear Export: The Journey of HIV-1 RRE-Containing RNAs

Although it is well established that the primary effect of Rev is to promote the nuclear export of RRE- containing HIV RNAs, many aspects of this process are still poorly understood. Rev integrates several cellular posttranscriptional mechanisms, such as mRNA splicing, RNA stability and nucleocytoplasmic transport. However, Rev does not possess any enzymatic activity and functions as an adaptor between RRE-containing RNAs and cellular factors. Hence, the activity of Rev is to engage in a promiscuous menage-a-trois with RRE and cellular proteins to shift the equilibrium from spliced/RRE-containing viral RNAs towards export of the latter.

The journey of the viral RNA starts at the transcription site. As soon as RNA is synthesized by RNAPII, its post-transcriptional processing begins through binding of proteins to the nascent RNA. These include the splicing factors described previously. For example, the splicing factor SF2/ASF appears enriched at the transcription site on nascent RNA (Figure 1C). In addition, INS RNA sequences and associated factors compete with splicing and retain unspliced and partially spliced RNA that are unstable in the nucleus in the absence of Rev. When Rev is present in the nucleus, the viral RRE-containing RNAs are stabilized and exported, but where does Rev recognize its substrate?

Studies of Cochrane and co-workers found that Rev could only export newly synthesized HIV RNA indicating that Rev acts on nascent transcripts rather than on downstream pathways engaged in splicing or RNA degradation [111]. Another indirect demonstration of Rev acting co-transcriptionally comes from early studies on the transactivation potential of Tat-Rev hybrids tethered to TAR-less constructs through association with the RRE [112,113]. Therefore, rather than forming a nuclear storage compartment for viral pre-mRNA where Rev would act, as it has been previously proposed [51,53,114], HIV-1 pre-mRNA appears to be associated with Rev at the site of transcription from where it is further exported. However, direct evidence of Rev at the transcription site is still missing since most studies concerning Rev subcellular localization did not carefully investigate the nascent proviral RNA at the same time [114,115,116,117,118,119]. 

Rev recognizes RRE as a monomer [120] and was quickly found to form oligomers on the RNA [20,121,122,123,124,125]. The crystal structure for Rev and a structural model to describe how Rev binds to the RRE, oligomerizes, and forms the RNA-protein complex which serves as the export substrate for Crm-1 recently became available [126,127,128,129]. This has been an important achievement, particularly for the rational design of drugs that could interfere with these interactions, but how Rev/RRE binding promotes export remains obscure. Actually, as pointed out in the work from Frankel’s laboratory, multimerization per se may not be used to recruit multiple Crm-1 molecules since steric hindrance would not allow more than one or two molecules of Crm-1 to bind the complex [126]. This implies that Rev oligomerization is needed to enhance RNA-binding affinity and not necessarily to recruit additional Crm-1 molecules. This model suggests that viral RNA is bound to one face of the Rev oligomer with Crm1 at the opposite face, providing a simple architecture to facilitate interactions with the nuclear pore and promote RNA export. Very recent experiments, conducted by a gain-of-function approach in the cellular environment, further confirmed the requirement of Rev oligomerization for nuclear export of unspliced and incompletely spliced RNA [130]. However, it appears that the function of the Rev-oligomer/RRE complex goes beyond the simple recruitment of Crm-1 molecules and may be important for the assembly of additional host factors like hRIP, eIF5A, Sam68 and RNA helicases which, in addition to a putative role in translocating the RNA across the nuclear pore, could also serve in the remodeling of the RRE during Rev oligomerization [131].

The journey of viral RNAs bound to Rev in the nucleus finally ends at the nuclear pore where the Crm-1/RRE/Rev complex is translocated to the cytoplasm as described previously. Little is known about this process. Certain nucleoporins were shown to be associated to the Crm-1/RRE/Rev complex and may be involved in the initial steps of pore recognition, while helicases such as DDX3 may assist RNA translocation. The kinetics of the process is also unknown, the application of single cell live imaging could eventually address this issue [132,133].

## 6. Conclusions

The mechanism of Rev function in the nucleus is likely to be governed by kinetic competition. The rate of viral RNA biogenesis determines the amount of unspliced and partially spliced RNAs that are formed at the transcription site. In fact the high transcription rate observed for the HIV-1 provirus also resulted in the presence of a large fraction of unspliced RNAs at the transcription site possibly indicating an effect of RNAPII velocity on splicing efficiency as was recently proposed [33,34]. This RNA is either spliced or quickly degraded by the action of INS-binding factors and unknown nucleases. However, increasing concentrations of Rev in the nucleus compete with the splicing/degradation pathway by preserving the RRE-containing RNA for export. Levels of Rev in the nucleus are determined by the cell type and are probably an effect of the efficiency of the nuclear import/export pathway for Rev. Finally, the Rev/RRE complex must engage Crm-1, again a nuclear shuttling protein, for export. 

Several questions remain unanswered. What are the cellular factors that determine the fate of unspliced HIV-1 RNA in the absence of Rev? Does Rev localize on nascent RNA? Where does Rev engage Crm-1? Future studies on HIV-1 Rev will need to address these issues with a combination of standard molecular biology techniques and the new tools that are available for high resolution live cell analysis [29,134]. 
